# Separation of Anatomical Contributions in MRSI Using a Non‐Negative Geographically Weighted Regression Model

**DOI:** 10.1002/nbm.70424

**Published:** 2026-10-22

**Authors:** Clément Giraud, Arnaud Poinas, Carole Guillevin, Rémy Guillevin

**Affiliations:** ^1^ Department of Imaging University Hospital Center of Poitiers Poitiers France; ^2^ DACTIM‐MIS team, Laboratory of Mathematics and Applications Labcom I3M Poitiers France

**Keywords:** brain metabolites, geographically weighted regression, magnetic resonance spectroscopy imaging, neuroimaging, partial volume effect, tissue segmentation

## Abstract

Magnetic resonance spectroscopic imaging (MRSI) is a non‐invasive neuroimaging tool that maps brain metabolites, providing insights into brain physiology and pathology. However, clinical adoption remains limited due to low spatial resolution and partial volume effects, where acquired signals detect metabolism from different tissue types, preventing clear identification of tissue‐specific metabolic patterns. We developed a statistical framework based on non‐negative geographically weighted regression (NNGWR) designed to overcome these limitations. The method integrates tissue partial volumes with MRSI data using spatially adaptive regression to interpolate metabolite levels across the imaging volume while separating distinct tissue contributions. Importantly, our model accounts for spatial heterogeneity within tissues rather than assuming metabolic uniformity, providing more physiologically realistic estimates. Simulations using synthetic ground truth data show that our method consistently achieves a lower median absolute percentage error than cubic spline and zero‐filling interpolations, especially under high noise or low‐resolution conditions. A total of 120 MRSI datasets were acquired at 3 T from a cohort of 36 healthy volunteers to assess predictive performance. To handle real‐world data, we implemented a robust reweighting scheme that addresses variable spectral quality by progressively downweighting degraded data rather than discarding it, balancing data retention with model stability. Application to a glioblastoma clinical case demonstrated successful separation of metabolite ratios across healthy, tumour and necrotic tissues. The model identified intratumoural metabolic heterogeneity and provided local confidence for transparent clinical interpretation. By decomposing metabolic contributions by tissue type and accounting for natural spatial variations, the approach enables improved brain tumour characterisation. This model is applicable to other neurological conditions where tissue‐specific metabolic alterations serve as disease biomarkers, helping both clinical diagnostics and research into brain pathophysiology.

## Graphical abstract

Non‐negative geographically weighted regression separates tissue‐specific metabolic signals in MRSI while correcting for partial‐volume effects. Coupled with anatomical segmentation, it enables spatial interpolation of metabolite maps with realistic intratissue heterogeneity. Validation on 120 healthy‐volunteer datasets and a glioblastoma case shows robust tissue discrimination and improved detection of metabolic heterogeneity.

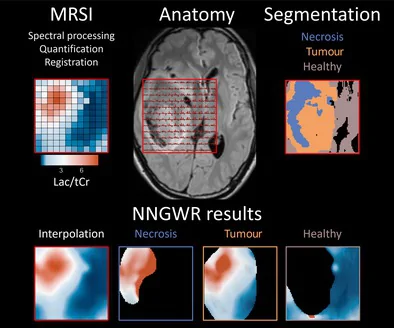

**Abbreviations**
LOOCV: leave‐one‐out cross‐validation
MRSI: magnetic resonance spectroscopic imaging
NNGWR: non‐negative geographically weighted regression
PSF: point spread function
SLIM: spectral localisation by imaging
SRF: spatial response function

## 1 Introduction

MRSI is a non‐invasive imaging technique that enables the spatial mapping of brain metabolites across a two‐ or three‐dimensional grid. By acquiring multiple magnetic resonance spectroscopy (MRS) voxels simultaneously, MRSI provides metabolic information from different brain locations, offering potential insights into neurological [1] and psychiatric disorders [2, 3].

However, to achieve sufficient spectral sensitivity and an adequate signal‐to‐noise ratio (SNR) within a clinically feasible acquisition time, MRSI requires larger voxel volumes. This results in a low spatial resolution, which in turn leads to partial volume effects, and limits the broader clinical and research applications of this technique [4].

Some approaches have incorporated anatomical information for super‐resolution MRSI reconstruction. An early representative method is the spectral localisation by imaging (SLIM) algorithm [5], which assumes homogeneous spectra within predefined anatomical compartments. Although effective in some settings, this assumption can lead to inaccurate metabolite quantification in regions exhibiting substantial spatial heterogeneity.

More recently, deep learning‐based approaches have been proposed for MRSI super‐resolution [6, 7]. However, their applicability is limited by the need for large, carefully curated training datasets and by the reliance on simulated high‐resolution ground truth because true high‐resolution MRSI acquisitions remain impractical [4]. Moreover, such models often provide limited voxel‐wise uncertainty estimates and offer restricted interpretability with respect to region‐specific metabolic behaviour. Their performance is further affected by variations in acquisition protocols, scanner hardware and spectroscopic preprocessing pipelines, which can hinder generalisation across sites and increase the computational burden associated with model retraining and deployment.

Despite these advances, existing MRSI reconstruction and analysis methods do not offer a clinically applicable framework able to separate metabolic information into spatially heterogeneous compartments. To overcome this challenge, we propose a statistical framework based on a non‐negative geographically weighted regression (NNGWR) model, which is well suited to the spatial sparsity of MRSI grids and the expected local variability of brain metabolism. Rather than reconstructing a higher‐resolution grid, our approach interpolates metabolic levels across the volume of interest (VOI) while simultaneously separating the interpolated values according to each partial volume contributions. Furthermore, this approach allows metabolic distributions to vary within anatomical compartments, providing a more physiologically meaningful representation.

Real‐world clinical data are subject to variations in spectral quality. They may arise from magnetic field inhomogeneities, coil sensitivity, subject motion, voxel positioning near tissue interfaces with air or lipids, and chemical shift displacement error (CSDE) [8, 9]. To address the challenge of fitting models to data with heterogeneous quality, we introduce a robust reweighting method. By systematically downweighting degraded spectra, this approach enables the model to rely preferentially on higher‐quality observations. Furthermore, this method is generalisable to most regression models and provides a smoother alternative to the hard‐thresholding practices commonly used in MRS quality control [10].

The validation of such methods is inherently challenging, because no internal reference standard is available to validate super‐resolution and interpolation methods in MRSI [4]. Therefore, the proposed framework is evaluated on a healthy cohort, allowing the model's predictive performance along with the reweighting scheme efficacy to be assessed under physiologically standard conditions. Additionally, the behaviour of model performance is analysed on simulated data under different acquisition conditions, particularly with respect to spatial resolution and acquisition noise.

Interpretability is particularly critical in clinically heterogeneous pathologies such as glioblastoma, where metabolic profiles differ markedly between necrotic core, infiltrative tumour tissue and surrounding healthy brain [11]. We therefore apply the proposed model in a glioblastoma clinical case study, focusing on the discrimination of metabolite ratios across these brain tissues to illustrate its potential for clinically meaningful and spatially resolved metabolic interpretation.

## 2 Theory

### 2.1 MRS Signal Quantification

An MRS signal is a complex, exponentially damped oscillation generated by the precession of nuclear spins in a magnetic field. This signal contains multiple frequency components, allowing to identify different metabolites, with their amplitudes, linewidths and phases influenced by relaxation effects and local magnetic field inhomogeneities.

In the frequency domain, the unperturbed continuous MR spectrum $S^{*}(r,\nu )$ at frequency ν and location r may be modelled as a linear combination of basis sets ${\left\{\phi_{i}(\nu )\right\}}_{i=1,\ldots ,M}$ and a baseline function $B^{*}(r,\nu )$

(1) $$S^{*}(r,\nu )=\overset{M}{\underset{i=1}{\sum }}A_{i}^{*}(r)\phi_{i}(\nu )+B^{*}(r,\nu ),$$

where $A_{i}^{*}(r)$ is proportional to the concentration of the i‐th metabolite at location r.

### 2.2 MRSI Volume Selectivity

We consider that we observe MR spectra localised in a phase encoded voxel grid with anatomical image voxels localised in a common reference frame. We denote by n the number of voxels. Compared with the unperturbed signal $S^{*}$, the measured signal S in a given MRS voxel can be seen as a convolution of $S^{*}$ with the spatial response function (SRF) observed on the voxel's centre denoted by $r\in {\mathbb{R}}^{3}$:

$$S(r,\nu )=(S^{*}(\cdot ,\nu )*\text{SRF})(r),$$

where ∗ is the convolution operator

(2) $$(S^{*}(\cdot ,\nu )*\text{SRF})(r)=\int_{\;{\mathbb{R}}^{3}}S^{*}(x,\nu )\text{SRF}(r-x)dx.$$

The SRF describes, for a single voxel, in image space, the weighted contribution to its signal from each point in object space [4, 12]. We can deduce its shape from the point spread function (PSF) and the eventual filter applied to the k‐space (e.g., Hamming filtering). This kind of modelling appears in most high‐resolution MRSI studies [4, 13]. Applying quantification models such as (1) to the observed signal S(r,ν) gives

$$S(r,\nu )=\overset{M}{\underset{i=1}{\sum }}A_{i}(r)\phi_{i}(\nu ;\theta_{i,r})+B(r,\nu ),$$

where $\theta_{i,r}$ denotes the parameters of the i‐th basis set estimated for the MR spectrum in the voxel centred at r. Combining this model with Equations 1 and (2) tells us that

(3) $$A_{i}(r)=\left(A_{i}^{*}*\text{SRF}\right)(r),$$

so that the coefficient $A_{i}(r)$ estimated while quantifying the MRSI signal is proportional to the amount of the i‐th metabolite weighted by the SRF centred at r. Ideally, the SRF would correspond to the nominal voxel. Although, in practice, the SRF is usually broader than the nominal voxel, meaning that some metabolites outside of the voxel also have some contribution to $A_{i}$. This phenomenon, commonly referred to as voxel bleeding, induces spatial dependencies between adjacent voxels. Such spatial dependencies will be exploited in the following model.

### 2.3 NNGWR Model

#### 2.3.1 Model Construction

Our main objective is to increase the MRSI grid spatial resolution by proposing a spatial prediction of the observed concentrations $A_{i}(r)$. We assume that their behaviour depends on the underlying anatomical structure, such as the presence of grey matter (GM), white matter (WM), cerebrospinal fluid (CSF) and other possible region of interest, like brain lesions [14, 15]. Therefore, our main assumption is that we can separate the brain into p regions in which the quantity of a given metabolite is slowly spatially varying. We then write

$$A_{i}^{*}(r)=\overset{p}{\underset{j=1}{\sum }}A_{i}^{*}(r)\cdot \delta_{j}(r),$$

where $\delta_{j}$ is the binary mask of value 1 when r is in the j‐th brain region, and 0 otherwise. This leads to the following decomposition of (3) for each brain region

$$A_{i}(r)=\overset{p}{\underset{j=1}{\sum }}\left(\left(A_{i}^{*}\cdot \delta_{j}\right)*\text{SRF}\right)(r).$$

This model can be rewritten as

(4) $$A_{i}(r)=\overset{p}{\underset{j=1}{\sum }}\beta_{i,j}(r)\cdot \left(\delta_{j}*\text{SRF}\right)(r),$$

where

(5) $$\beta_{i,j}(r)=\left[\left(A_{i}^{*}\cdot \delta_{j}\right)*\text{SRF}/\delta_{j}*\text{SRF}\right](r),$$

corresponds to the average i‐th metabolic concentration inside the j‐th tissue in the neighbourhood of location r. This motivates the use of a non negative constraint on $\beta_{i,j}(r)$. Compared with SLIM‐based methods, this coefficient separates contribution of each compartment without assuming metabolic homogeneity.

The SRF is a unit‐mass function concentrated around the origin. It follows that, for any point r not too close to the border of the voxel grid, we have

(6) $$\overset{p}{\underset{j=1}{\sum }}\delta_{j}*\text{SRF}(r)\approx 1,$$

meaning that $\delta_{j}*\text{SRF}(r)$ corresponds to the proportion of the j‐th tissue in the neighbourhood of location r weighted by the SRF.

The model (4) is a linear regression between $A_{i}(r)$ and each $\left(\delta_{j}*\text{SRF}\right)(r)$ for j in {1,…,p}, where each associated coefficient $\beta_{i,j}(r)$ does depend on the location of the observed metabolite concentration, but is assumed to be slowly varying. Such a setting is defined as a geographically weighted regression (GWR) model. The addition of a constraint of non‐negativeness for each $\beta_{i,j}$ makes it an NNGWR model. Such a model enables the estimation of $A_{i}$ at any position in the VOI while integrating spatial dependencies and anatomical characteristics. The estimation of each $\beta_{i,j}$ also provides additional information as it represents the contribution of each segmented region of the brain to the amount of metabolite observed.

The present work focuses on metabolite ratios as they are generally preferred to absolute quantification in clinical applications [16]. Given a pair $m=(i_{1},i_{2})$ of metabolite indices, we define $A_{m}^{*}=A_{i_{1}}^{*}/A_{i_{2}}^{*}$ as the corresponding metabolite ratio. It can be represented at the instrument's resolution by

(7) $$A_{m}(r)=\frac{A_{i_{1}}(r)}{A_{i_{2}}(r)}=\frac{\left(A_{i_{1}}^{*}*\text{SRF})(r\right)}{\left(A_{i_{2}}^{*}*\text{SRF})(r\right)}.$$

The spatial prediction of $A_{m}$ can be achieved by individually predicting the observations $A_{i_{1}}(r)$ and $A_{i_{2}}(r)$ using a separate NNGWR model (4) for both metabolites. The average metabolite ratio inside the j‐th tissue in the neighbourhood of location r:

$$\frac{\left(\frac{A_{i_{1}}^{*}}{A_{i_{2}}^{*}}\cdot \delta_{j}\right)*\text{SRF}(r)}{\delta_{j}*\text{SRF}(r)},$$

can be approximated (see Appendix A for the mathematical details) by

(8) $$\frac{\beta_{i_{1},j}(r)}{\beta_{i_{2},j}(r)}=\frac{\left(A_{i_{1}}^{*}\cdot \delta_{j})*\text{SRF}(r\right)}{\left(A_{i_{2}}^{*}\cdot \delta_{j})*\text{SRF}(r\right)},$$

and consequently be estimated by ${\hat{\beta }}_{i_{1},j}(r)/{\hat{\beta }}_{i_{2},j}(r)$. However, this estimate becomes unreliable when the denominator approaches zero. This occurs when we observe a low amount of the $i_{2}$‐th metabolite, combined with a small occurrence of the j‐th tissue, around r. Therefore, it is expected that (8) cannot be reliably estimated under these conditions, as it becomes difficult to assess the local contribution of a tissue that is nearly absent.

#### 2.3.2 Model Fitting

The calibration process in GWR assumes that data observed near a location r have more influence in the estimation of $A_{i}^{*}(r)$ than data that are farther [17]. This motivates the introduction of a weight vector between a location r and the voxel centres $r_{1},\ldots \;,r_{n}$ we define by

(9) $$W(r)=\mathrm{diag}\left({\left\{w_{j}\;K_{h}\left(d(r,r_{j})\right)\right\}}_{j=1}^{n}\right),$$

a diagonal matrix calculated at r where $K_{h}$ is a kernel function with bandwidth h weighting the distance between r and the j‐th voxel centroid, and $w_{1},\ldots \;,w_{n}$ are a set of weights associated with each voxel and used to downweight spectra of poor quality (see Section 2.3.3). We estimate the coefficients $\beta_{i,j}(r)$ in (4) at a given location r by solving the following constrained least squares problem

(10) $$\begin{array}{cc} {\hat{\beta }}_{i}(r)= & \underset{\beta \in {\mathbb{R}}^{p}}{\text{argmin}}\|X\beta -y{\|}_{W(r)}^{2}+\lambda \|\beta {\|}_{2}^{2}\\ & \text{s.t.}\;\beta_{j}\geq 0\;\forall j\in \{1,\ldots ,p\}. \end{array}$$

Here, ${\hat{\beta }}_{i}(r)$ denotes the vector of ${\hat{\beta }}_{i,j}(r),\;X$ is an n×p matrix containing the segmented brain tissues fractions (6) weighted by the SRF and $y={\left(A_{i}(r_{j})\right)}_{1\leq j\leq n}$ is a vector containing the observed metabolic concentrations in each voxel. The norm $\|\cdot {\|}_{W(r)}$ in (10) corresponds to the Euclidean norm weighted by the elements of W(r). We also introduce in (10) a ridge regularisation term with parameter λ. This regularisation not only mitigates multicollinearity but also prevents coefficient inflation where certain tissue fractions are near zero.

It is common practice when fitting a GWR model to adjust the bandwidth parameter h by minimising the corrected Akaike information criterion (AICc) [18], but the constraint of non‐negativity prevents its computation. Therefore, we use the leave‐one‐out cross‐validation (LOOCV) with row standardisation [19], to prevent individual data points from disproportionately affecting the choice of bandwidth. Once the bandwidth h is set, the regularisation parameter λ is adjusted with the same approach. Such model allows for prediction and therefore it can generate an image of arbitrary dimension of an estimate of $A_{i}^{*}(r)$ and $\beta_{i}(r)$.

#### 2.3.3 Robust Reweighting

Expert consensus generally recommends excluding from analysis any spectrum that does not meet predefined quality criteria using a hard‐thresholding approach [10]. However, spectral quality metrics such as Cramér–Rao lower bound (CRLB), full width at half maximum (FWHM) or SNR can vary substantially across the MRSI VOI [20], reflecting spatially dependent factors including magnetic field inhomogeneities, coil sensitivity, motion and partial‐volume effects [8, 9]. As a consequence, rigid exclusion criteria may lead to excessive data loss or biased spatial sampling.

To mitigate this, we propose a method inspired by the robust GWR framework [17] (section 3.4), in which each MRS voxel is weighted according to its spectral quality rather than being binary included or excluded. This approach allows voxels with reduced but acceptable data quality to contribute proportionally to the analysis.

In the present work, the weights associated to spectral quality used in (9) are based on the bisquare kernel, which has compact support and provides a smooth transition between unpenalised and discarded spectra

(11) $$\begin{array}{c} \;w_{j}=w_{j}^{\text{SNR}}w_{j}^{\text{FWHM}},\\ \;w_{j}^{\text{SNR}}=\left\{\begin{array}{ll} 1, & \;\text{if}\;{\text{SNR}}_{j}\geq M_{\text{SNR}},\\ {\left(1-{\left(\frac{{\text{SNR}}_{j}-M_{\text{SNR}}}{M_{\text{SNR}}-m_{\text{SNR}}}\right)}^{2}\right)}^{2}, & \;\text{if}\;m_{\text{SNR}}\leq {\text{SNR}}_{j}<M_{\text{SNR}},\\ 0, & \;\text{otherwise}, \end{array}\right.\\ w_{j}^{\text{FWHM}}=\left\{\begin{array}{ll} 1, & \text{if}\;{\text{FWHM}}_{j}\leq m_{\text{FWHM}},\\ {\left(1-{\left(\frac{{\text{FWHM}}_{j}-m_{\text{FWHM}}}{M_{\text{FWHM}}-m_{\text{FWHM}}}\right)}^{2}\right)}^{2}, & \text{if}\;m_{\text{FWHM}}<{\text{FWHM}}_{j}\leq M_{\text{FWHM}},\\ 0, & \text{otherwise}, \end{array}\right. \end{array}$$

where $M_{\text{SNR}}$ and $m_{\text{FWHM}}$ denotes the thresholds between unpenalised and penalised spectra and $m_{\text{SNR}}$ and $M_{\text{FWHM}}$ denotes the thresholds between discarded and penalised spectra. This choice ensures robustness by limiting the influence of low‐quality observations while avoiding abrupt thresholding effects. The lower and upper thresholds can be defined arbitrarily or defined adaptively from the empirical distribution of SNR and FWHM within the VOI using robust statistics, such as

$$\begin{array}{l} \;m_{\text{SNR}}=Q_{1}^{\text{SNR}}-1.5\left(Q_{3}^{\text{SNR}}-Q_{1}^{\text{SNR}}\right)\;\\ \;M_{\text{SNR}}=Q_{3}^{\text{SNR}}\;\\ m_{\text{FWHM}}=Q_{1}^{\text{FWHM}}\;\\ M_{\text{FWHM}}=Q_{3}^{\text{FWHM}}+1.5\left(Q_{3}^{\text{FWHM}}-Q_{1}^{\text{FWHM}}\right).\; \end{array}$$

Here, $Q_{1}$ and $Q_{3}$ are the first and third quartiles respectively. They are used to define data‐driven and objective thresholds that adapt to the quality variability between MRS sequences and applications. As a result, spectra are progressively penalised according to their deviation from the top quartile of observed quality metrics. Spectra identified as extreme outliers, either exhibiting abnormally low SNR or excessively large FWHM, are assigned zero weight and excluded from the analysis. This weighting strategy is applied consistently across all models and its impact is assessed on the healthy cohort. The weight function as defined in (11) is applicable across all metabolite models, ensuring a consistent evaluation framework. However, it can be specified for a single metabolite by adding a term based on its CRLB.

An advantageous property of this reweighting scheme is that, when the spectral quality metrics are locally similar, the weights tend to be approximately equal,

$$w_{1}=w_{2}=\ldots =w_{n}\neq 0.$$

In this case, the weighting matrix in (9) effectively reduces to the conventional spatial weighting matrix used in GWR [17]. Consequently, the model keeps its standard behaviour when spectral quality is homogeneous.

## 3 Methods

### 3.1 Data Acquisition and Processing

Both a cohort of healthy volunteers [21] and a clinical case with a histologically confirmed glioblastoma, for which written informed consent was obtained, were acquired at 3 T using the same MRS protocol detailed in Table B1 [10]. The study was approved by the national ethics committee, and all participants provided written informed consent after being fully briefed on the study's objectives, benefits and potential risks.

^1^H‐MRSI data were collected using a 2D chemical shift imaging (CSI) semi‐localized by adiabatic selective refocusing (sLASER) sequence (echo time [TE] = 135 ms) over a VOI of 82.5×82.5×20 mm^3^ with a 12×12 acquisition matrix, resulting in a nominal voxel resolution of 6.875×6.875×20 mm^3^. We focus our analysis on the following commonly used metabolite ratios: total choline: phosphocholine + glycerophosphocholine (tCho) to total *N*‐acetylaspartate: *N*‐acetylaspartate + *N*‐acetylaspartylglutamate (tNAA), tNAA to total creatine: creatine + phosphocreatine (tCr), tCho to tCr, and lactate (Lac) to tCr [11].

The MRSI were registered to the T1‐weighted MPRAGE. First, the images were resampled to an isotropic resolution of 1mm. Then, brain tissue segmentation was performed using the FMRIB Software Library (FSL) package [22]. CSF masks were removed from models because we assume that the studied metabolite ratios are close to zero in the CSF.

MR spectra were processed in accordance with expert consensus recommandations [15], using the R software [23] Version 4.1.0 and the SPANT library [24] Version 3.8.0. Quantification was then performed using LCModel [25] with a basis set of brain metabolites simulated on MATLAB. MRS protocol details are given in Table B1.

### 3.2 Evaluation Procedure

We evaluate the model using two complementary approaches: numerical simulations and a study of a healthy cohort. The simulations provide an assessment of performance under varying acquisition conditions by modulating spatial resolution and noise levels. Furthermore, the study of the healthy cohort serves to evaluate the model's overall reliability and the effectiveness of the robust reweighting method.

Distributions mean and standard deviation are reported as μ±σ. For both simulation and cohort studies, a Gaussian kernel was selected in (9) to spatially weight the data, which is defined as

$$K_{h}(d)=\exp\left(-\frac{d^{2}}{h^{2}}\right).$$

#### 3.2.1 Simulation Study

We simulated different CSI acquisition conditions using synthetic data, following the pipeline detailed below and schematised in Figure 1. The performance of the proposed model was benchmarked against conventional spatial interpolation methods, including cubic spline and zero‐filling techniques [26], using upsampling factors of × 2 and × 16. The × 2 factor was selected to align with established recommendations in clinical proton MRS [16], whereas the × 16 factor and cubic spline interpolation were included to reflect approaches commonly implemented in radiological imaging software [4]. For computational efficiency, all simulations were performed in 2D.

1. Brain tissues segmentation:
A 2D slice of the segmented MNI template brain [27] is randomly selected, providing a segmented brain map with distinct GM and WM regions.
2. Field simulation:
Two independent stationary isotropic Gaussian random fields are simulated to represent spatial variations in metabolite concentrations across GM and WM. Both fields share an exponential covariance function with a variance of 2 mM/kg and a range parameter of 10 mm. To reflect the distribution of *N*‐acetyl‐aspartate (NAA) concentration [28], region‐specific means are assigned: 20.55 mM/kg for GM and 13.75 mM/kg for WM. NAA concentration in the CSF is assumed to be negligible.
3. Sampling:
The simulated fields are combined and convolved with the SRF. The resulting field, which constitutes the synthetised ground truth, is sampled on a discrete grid, with parameters defined by the VOI dimensions and phase encoding matrix size. Gaussian white noise is added to simulate sources of data corruption that can arise during MRSI acquisitions.
4. Models fit:
The proposed model (4) and reference methods are each applied separately to the sampled field. The median absolute percentage error (MdAPE) values of all models are calculated and compared, as defined in (12).

**FIGURE 1:**
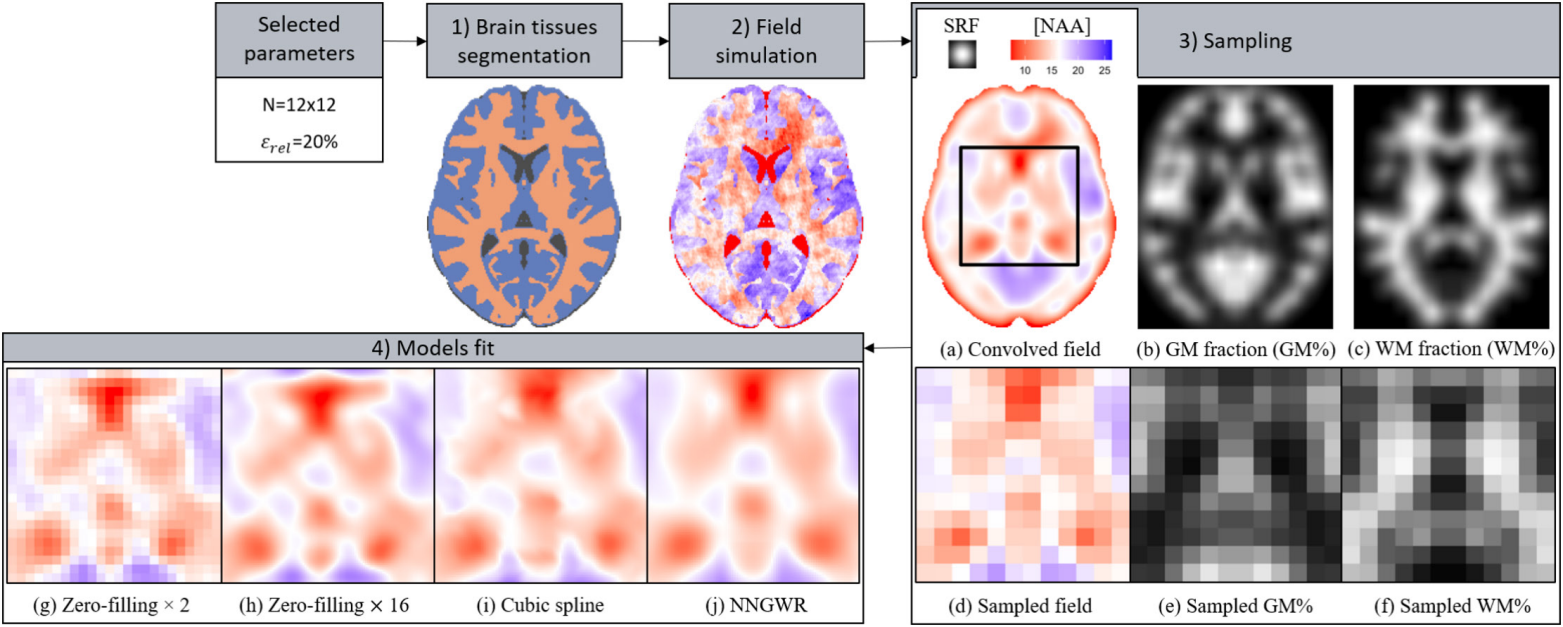
Simulation pipeline. (1) 2D segmented brain map with GM and WM masks. (2) Random field generated in GM and WM, representing NAA concentration. (a) Random field convolved with the SRF. (b, c) GM and WM masks convolved with the SRF to produce tissue fraction maps. (d) Convolved fields sampled on a discrete grid and corrupted with relative noise to simulate MRSI acquisition. (e, f) Corresponding brain tissue rates. Final interpolations using × 2 and × 16 zero‐filling (g, h), cubic spline (i), and the proposed model (j). Performance is evaluated using the MdAPE (12).

The validation criterion is the MdAPE, computed between the model predictions and the ground truth data

(12) $$\text{MdAPE}=\underset{i\in \Omega ,\;y_{i}\neq 0}{\mathrm{median}}\left(100\cdot \frac{\left|y_{i}-{\hat{y}}_{i}\right|}{\left|y_{i}\right|}\right),$$

where $y_{i}$ and ${\hat{y}}_{i}$ are the ground truth and predicted values at point i∈Ω. Here, Ω denotes the model prediction coordinates.

#### 3.2.2 Cohort Study

In GWR, its quality‐of‐fit is often assessed using the adjusted *R*‐squared [17]. However, this statistic cannot be computed as such due to the non‐negativity constraint on the coefficients not being taken into account (10). Instead, we propose to estimate it by LOOCV, which penalises overfitting and provides a more realistic estimate of predictive performance under this model constraints. The *R*‐squared estimate is computed by weighting the LOOCV prediction errors using (11) to account for the reweighting process, as follows:

$$R^{2}=1-\left(\frac{\overset{n}{\underset{i=1}{\sum }}w_{i}{\left(y_{i}-{\hat{y}}_{i}^{\left(-i\right)}\right)}^{2}}{\overset{n}{\underset{i=1}{\sum }}w_{i}{\left(y_{i}-\bar{y}\right)}^{2}}\right)$$

where ${\hat{y}}_{i}^{\left(-i\right)}$ is the prediction of the observation i obtained from a model trained without this observation and $\bar{y}$ is the overall mean of the observed values.

The impact of applying the robust reweighting scheme defined in (9) was evaluated by introducing controlled spectral perturbations before spectral quantification. For each MRSI acquisition in the cohort, to simulate heterogeneous MRSI spectral quality, spectral degradation was performed at the following voxel locations in the 12×12 grid: $\Omega_{2}=\{(4,4),(4,9),(9,4),(9,9)\}$. For these four voxels, we applied line‐broadening of 5 Hz and added a normally distributed noise to target half the original SNR.

The absolute percentage error from the original estimates ${\hat{\beta }}_{\text{ref}}$ was calculated, and the distributions of its median and maximum (13) were then compared between the reweighted and non‐reweighted models

(13) $$\begin{array}{cc} {\text{MdAPE}}_{\beta }=\underset{(i,j)\in \Omega_{2},\;{\hat{\beta }}_{\text{ref},i,j}\neq 0}{\mathrm{median}} & \left(100\cdot \frac{\left|{\hat{\beta }}_{\text{ref},i,j}-{\hat{\beta }}_{i,j}\right|}{\left|{\hat{\beta }}_{\text{ref},i,j}\right|}\right)\\ {\text{MxAPE}}_{\beta }=\underset{(i,j)\in \Omega_{2},\;{\hat{\beta }}_{\text{ref},i,j}\neq 0}{\max} & \left(100\cdot \frac{\left|{\hat{\beta }}_{\text{ref},i,j}-{\hat{\beta }}_{i,j}\right|}{\left|{\hat{\beta }}_{\text{ref},i,j}\right|}\right). \end{array}$$

## 4 Results

### 4.1 Simulations

A total of 1200 interleaved simulations were generated by processing 100 synthetic data samples, each centred within a 100 mm^2^ VOI, across 12 experimental settings defined by four phase‐encoding matrix sizes (8×8,10×10,12×12 and 14×14), and three white noise levels (10*%*,  20*%* and 30*%* of the convolved field standard deviation). The resulting performance distributions for the models trained on these simulations are summarised in Table 1.

**TABLE 1:** Comparison of MdAPE across different models and simulation settings.

		MdAPE (in %)			
Matrix resolution	Relative noise	NNGWR	Zero‐filling × 2	Zero‐filling × 16	Spline
	10%	1.64 ± 0.23	4.91 ± 0.71	7.74 ± 1.17	2.59 ± 0.38
8	20%	2.17 ± 0.30	5.24 ± 0.69	7.97 ± 1.16	3.06 ± 0.39
	30%	2.85 ± 0.48	5.69 ± 0.79	8.28 ± 1.20	3.78 ± 0.48
	10%	1.38 ± 0.19	3.90 ± 0.56	6.51 ± 1.00	1.64 ± 0.21
10	20%	1.81 ± 0.23	4.32 ± 0.56	6.81 ± 0.98	2.35 ± 0.29
	30%	2.33 ± 0.31	5.01 ± 0.66	7.30 ± 1.03	3.19 ± 0.45
	10%	1.33 ± 0.21	3.47 ± 0.49	5.85 ± 0.89	1.40 ± 0.17
12	20%	1.70 ± 0.19	4.05 ± 0.51	6.23 ± 0.90	2.20 ± 0.24
	30%	2.14 ± 0.30	4.80 ± 0.63	6.81 ± 0.96	3.14 ± 0.40
	10%	1.36 ± 0.19	3.33 ± 0.48	5.57 ± 0.83	1.26 ± 0.14
14	20%	1.64 ± 0.20	3.97 ± 0.50	5.98 ± 0.82	2.14 ± 0.22
	30%	2.04 ± 0.24	4.80 ± 0.52	6.67 ± 0.84	3.08 ± 0.34

Out of the 1200 sampled cases, our model outperformed zero‐filling interpolation and cubic spline in 89.66% of instances while achieving a lower average MdAPE in every setting except the most favourable one, where cubic spline slightly outperformed our model at matrix resolution 14 and relative noise of 10% (model MdAPE = 1.36±0.19, spline = 1.26±0.14). As illustrated in Figure 2, the NNGWR model performs better than the reference methods in the most challenging settings, particularly in settings with high noise and small grid sizes.

**FIGURE 2:**
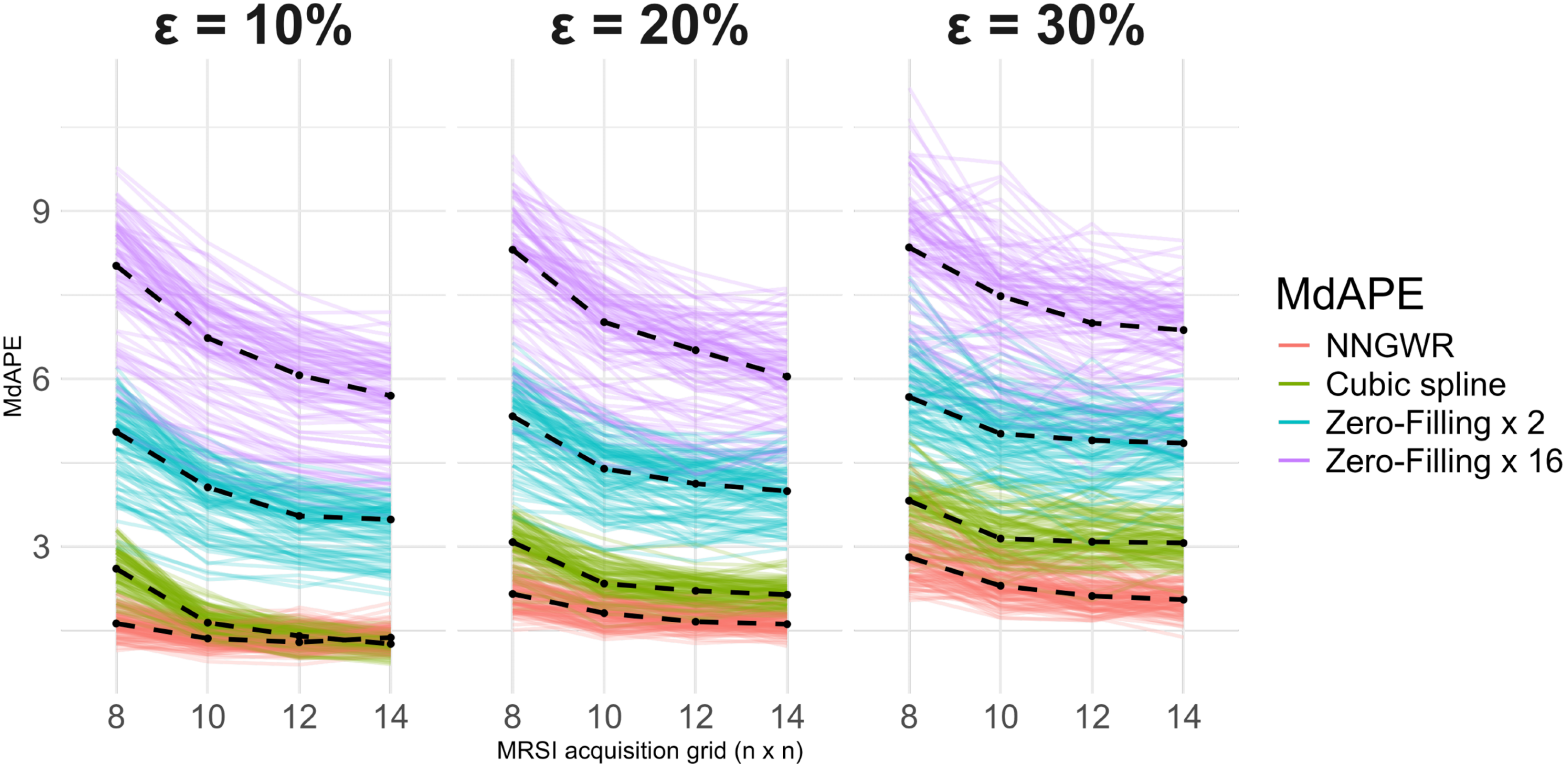
MdAPE of the NNGWR, spline and zero‐filling interpolations as a function of MRSI resolution grid size for each simulated random field.

The ability of the model to estimate simulated NAA concentrations within GM and WM partial volumes was assessed by computing the MdAPE for each tissue type separately. Across all simulation settings, the average MdAPE was 4.61±1.51, with values ranging from 2.95 to 7.40 (Table 2).

**TABLE 2:** MdAPE of the proposed model's estimates of simulated random fields by tissue type.

		MdAPE (in %)	
Matrix resolution	Relative noise	NNGWR (GM)	NNGWR (WM)
	10%	4.36 ± 1.17	4.83 ± 1.18
8	20%	5.48 ± 1.34	5.98 ± 1.40
	30%	6.77 ± 1.97	7.40 ± 2.05
	10%	3.45 ± 0.59	4.00 ± 0.74
10	20%	4.36 ± 0.96	4.91 ± 1.02
	30%	5.38 ± 1.10	5.79 ± 1.09
	10%	3.18 ± 0.52	3.68 ± 0.67
12	20%	3.81 ± 0.66	4.39 ± 0.80
	30%	4.84 ± 0.93	5.28 ± 1.00
	10%	2.95 ± 0.44	3.56 ± 0.60
14	20%	3.40 ± 0.52	3.98 ± 0.61
	30%	4.08 ± 0.71	4.69 ± 0.78

The simulated NAA concentration field shown in Figure 1 was used to map the percentage absolute error of the models across the VOI and is displayed in Figure 3. Notably, reference methods exhibits elevated error values within the CSF regions of the brain, because without anatomical information they are unable to make assumption on missing metabolic ratios. In contrast, our model is able to estimate the metabolic ratio in these regions, leading to a lower error.

**FIGURE 3:**
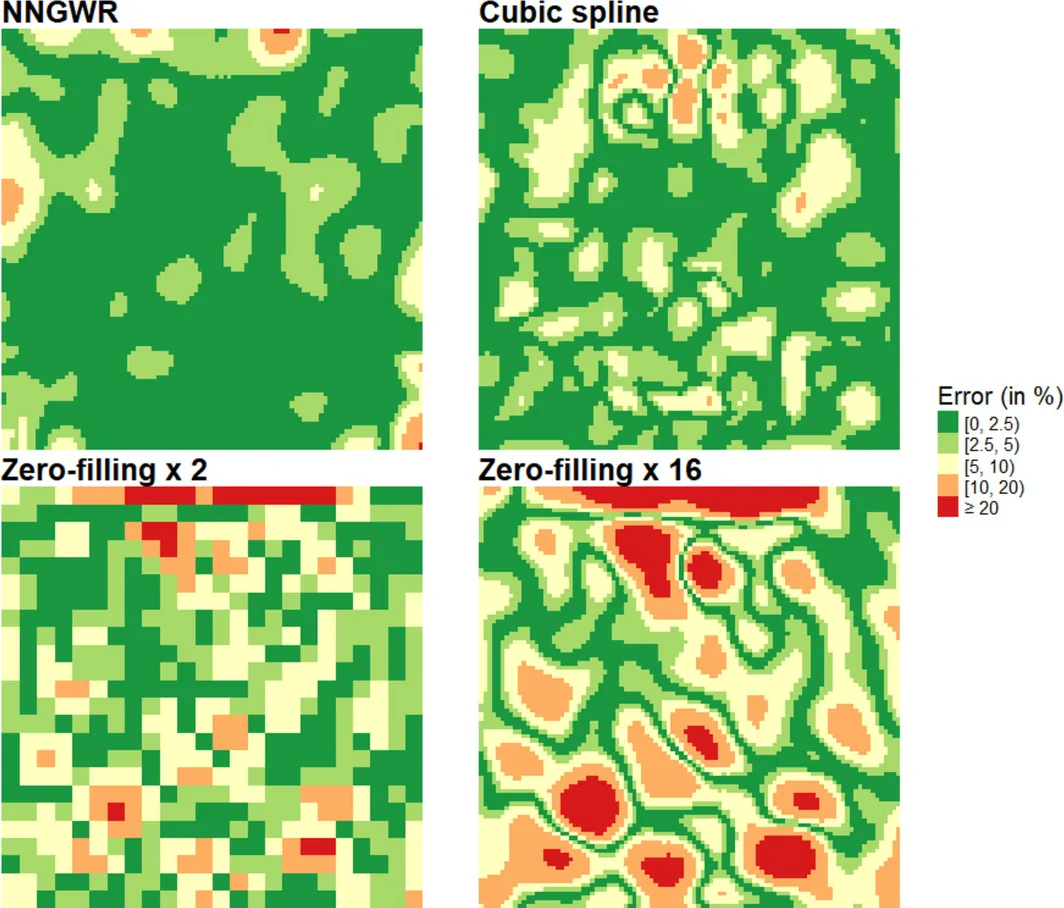
Example of models percentage absolute error across the VOI, trained with a sampled MRS grid of resolution 12×12 and a relative noise of 20*%*.

### 4.2 Cohort Evaluation

A total of 120 MRSI grids were acquired from 36 healthy volunteers (24 men and 12 women; mean age 28.9±5.1 years). These data are part of a cohort described in previous work [21], in which each volunteer underwent three 3 T MRI sessions. At each session, two CSI sLASER sequences were acquired, one in the semioval centre and one in the basal ganglia. To ensure consistency in performance comparisons, only grids with a resolution of 12×12 were included, resulting in more datasets from the semioval centre (*n* = 108) than from the basal ganglia (*n* = 12). The model's output is presented for a healthy volunteer from the cohort (see Appendix Section C).

The bandwidth parameter h follows a distribution of 1.15±0.17 for tCho, 1.12±0.19 for tNAA, 1.20±0.17 for tCr and 1.41±0.23 for Lac. Their distributions are significantly different (Kruskal–Wallis test, p<0.001).

The *R*‐squared values were distributed at 0.81±0.13 for tCho/tNAA ratio, 0.87±0.07 for tNAA/tCr ratio, 0.80±0.14 for tCho/tCr ratio and 0.37±0.21 for Lac/tCr ratio. When considering individual metabolites, *R*‐squared values were 0.86±0.05 for tCho, 0.92±0.04 for tNAA, 0.89±0.04 for tCr and 0.41±0.18 for Lac. Overall, *R*‐squared values were consistently high, except for the Lac and therefore the Lac/tCr models.

Within GM and WM, the distribution of mean metabolite ratios was, 0.15±0.05 and 0.15±0.04 for tCho/tNAA ratio, 1.99±0.56 and 2.35±0.39 for tNAA/tCr ratio, 0.29±0.07 and 0.34±0.07 for tCho/tCr ratio and 0.29±0.14 and 0.31±0.12 for Lac/tCr ratio, respectively.

To test the reweighting performance, specific spectra were synthetically degraded with 5 Hz line‐broadening and a twofold reduction in SNR. These targeted spectra exhibited the expected reduction in spectral quality, resulting in decreased quality weights (Table 3).

**TABLE 3:** Spectral quality assessment before and after applying perturbations.

Spectral perturbation	SNR	FWHM (ppm)	$w=w^{\text{SNR}}w^{\text{FWHM}}$
Before	24.21±8.90	0.124±0.022	0.86±0.13
After	12.30±4.45	0.133±0.015	0.16±0.16

Across all models, the application of robust weights reduced the ${\text{MdAPE}}_{\beta }$ from 3.06±1.47 to 1.11±0.92. Similarly, the distribution of ${\text{MxAPE}}_{\beta }$ improved significantly, shifting from 96.61±62.68 to 36.57±23.7.

The deviation from original estimates was consistently mitigated across all metabolites (Table 4), confirming the method's ability to maintain stable and accurate model estimation by downweighting the influence of low‐quality spectra.

**TABLE 4:** Distribution of estimates relative maximum (${\text{MxAPE}}_{\beta }$) and median (${\text{MdAPE}}_{\beta }$) deviation after spectral perturbation, evaluated with and without robust reweighting.

	${\text{MdAPE}}_{\beta }$ (in %)		${\text{MxAPE}}_{\beta }$ (in %)	
Robust reweighting	Yes	No	Yes	No
tCho	0.61±0.68	2.08±0.93	16.31±7.02	38.60±13.34
tNAA	1.25±1.12	3.19±1.64	48.29±34.75	110.78±54.66
tCr	0.91±0.86	2.77±1.03	26.45±13.51	64.73±23.33
Lac	0.84±0.78	3.45±1.52	53.47±34.99	94.97±54.98
tCho/tCr	1.01±0.65	2.58±1.00	21.13±11.70	64.00±37.70
tNAA/tCr	1.40±0.85	2.73±1.17	48.08±33.30	99.03±60.92
tCho/tNAA	1.38±1.03	3.54±1.67	42.86±28.66	257.00±181.55
Lac/tCr	1.53±0.93	4.28±1.44	59.75±33.58	171.46±109.09

### 4.3 Clinical Case Analysis

To display the interpretability of this model, we selected a patient with a glioblastoma in the central nervous system (Figure 4), which was annotated under multiple labels using an algorithm based on post‐contrast T1‐weighted, T1‐weighted, T2‐weighted and FLAIR images [29], then completed with FS. This resulted in a separation of the VOI in five regions: GM, WM, CSF, necrotic tumour core (denoted as *Core*) and surrounding non‐core tumour (denoted as *Tumour*). As the tumour occupies the majority of the VOI, the GM and WM were gathered under the label *Healthy* to get a more robust estimate. Masks are convolved with the SRF (Figure 5) to map the covariates of (4) across all the VOI.

**FIGURE 4:**
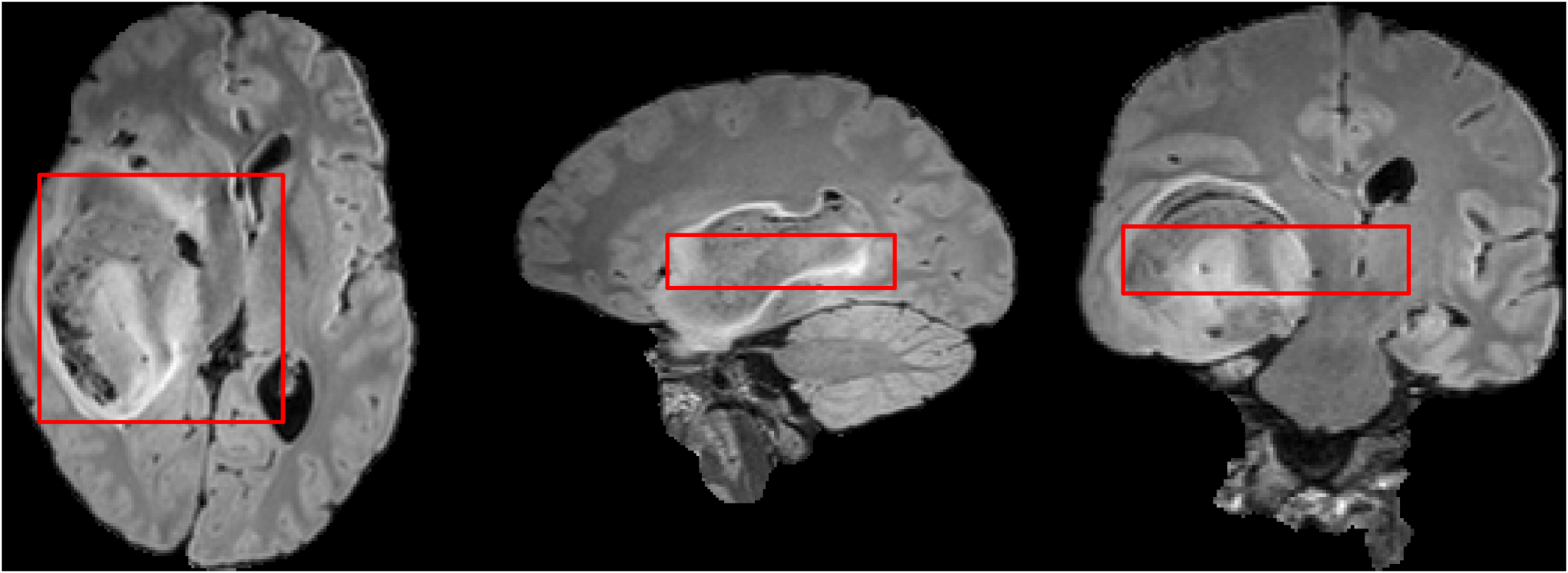
FLAIR image of a patient with a glioblastoma, along with the CSI VOI shown with red contour. This image was resampled to 1 mm isotropic prior to segmentation.

**FIGURE 5:**
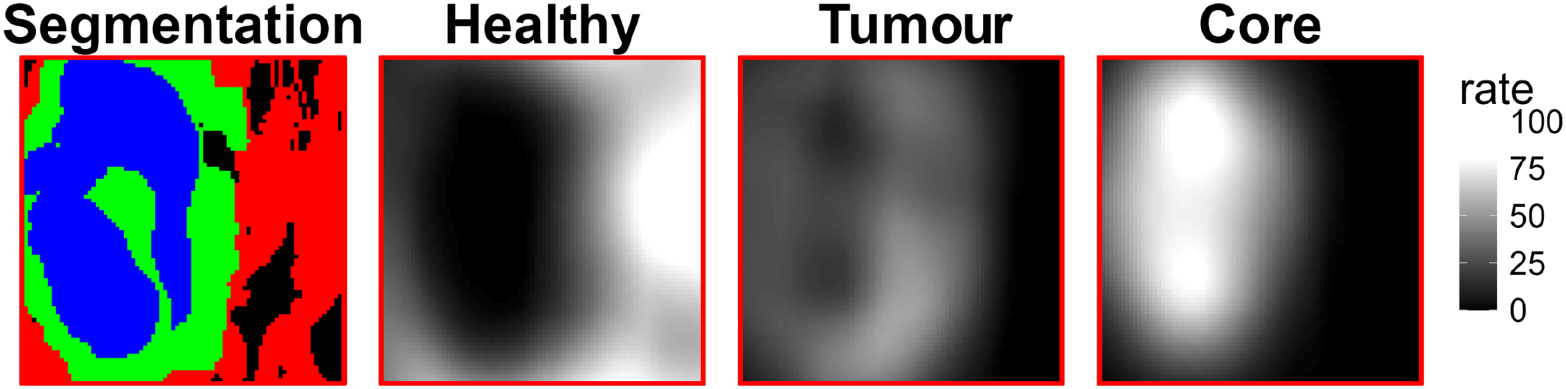
Segmented brain *Healthy* tissues (red), *Tumour* (green) and *Core* (blue). Each of these masks are then convolved with the SRF.

These segmentations allows us to identify three signals corresponding to voxels with the highest proportion of healthy tissue, non‐core and core tumour, respectively (Figure 6). Healthy spectra exhibit a normal metabolic profile, characterised by prominent tNAA and lower choline levels, with no detectable lactate. In contrast, tumour spectra show decreased tNAA, increased choline and the presence of a lactate peak. In areas of necrosis, tNAA is absent and lactate levels are markedly elevated.

**FIGURE 6:**
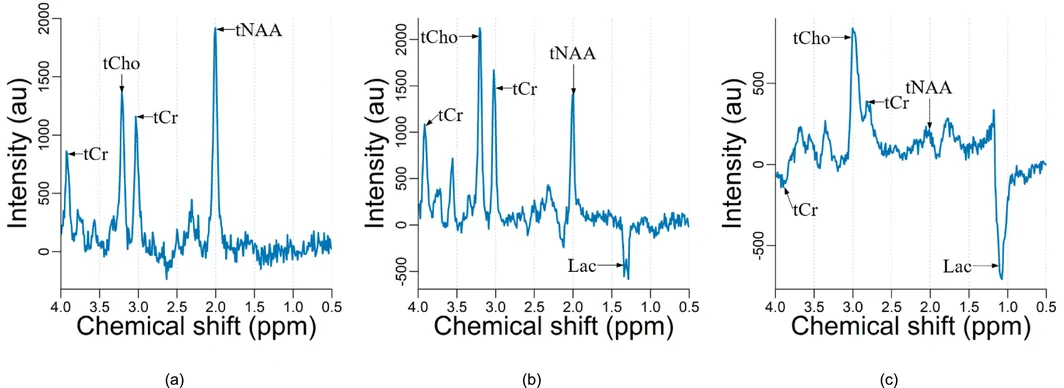
Spectra samples selected from voxels with the greater (A) healthy brain tissue rate, (B) tumour rate and (C) necrosis rate.

Spectral quality was calculated to compute the weights that will be used for robust regression. The resulting SNR and FWHM maps, along with their corresponding weights, are displayed in Figure 7. The most penalised data arise from the outer voxels, which generally exhibit lower SNR and increased FWHM. Notably, both criteria identified anomalous spectra at coordinates (1,2) and (1,3), for which the weights were set to zero and therefore excluded from the model fitting.

**FIGURE 7:**
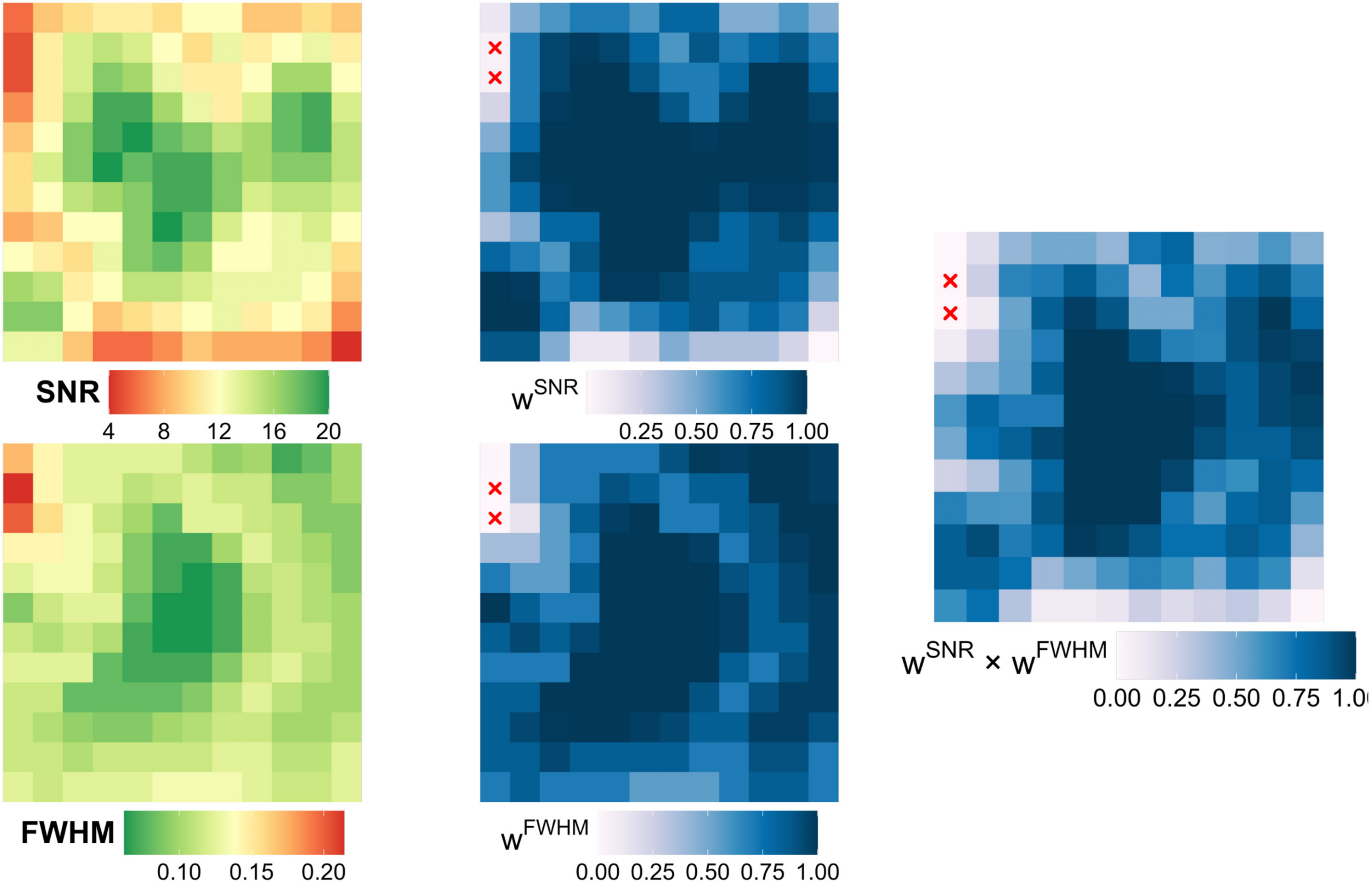
Spectra SNR and FWHM, along with their computed weights (11) and the final reweighting map used for the models' fit. Voxels at coordinates (1,2) and (1,3) were identified as outliers and their weights were set to zero.

Figure 8 displays the metabolite ratio grids obtained from the MRSI positioned on the glioblastoma (Figure 4), alongside the corresponding model predictions and the grid obtained after zero‐filling for comparison. For clarity, only a 2D slice of the middle of the VOI is shown. We recall that the interpolated value at a given location corresponds to the value of the metabolite ratio that would be observed from an MRS voxel centred at that location. Despite the poor signal quality at coordinate (1,2) (SNR = 5, FWHM = 0.214 ppm), which produces metabolite ratios spatially inconsistent with neighbouring voxels, the reweighting method successfully removed this outlier.

**FIGURE 8:**
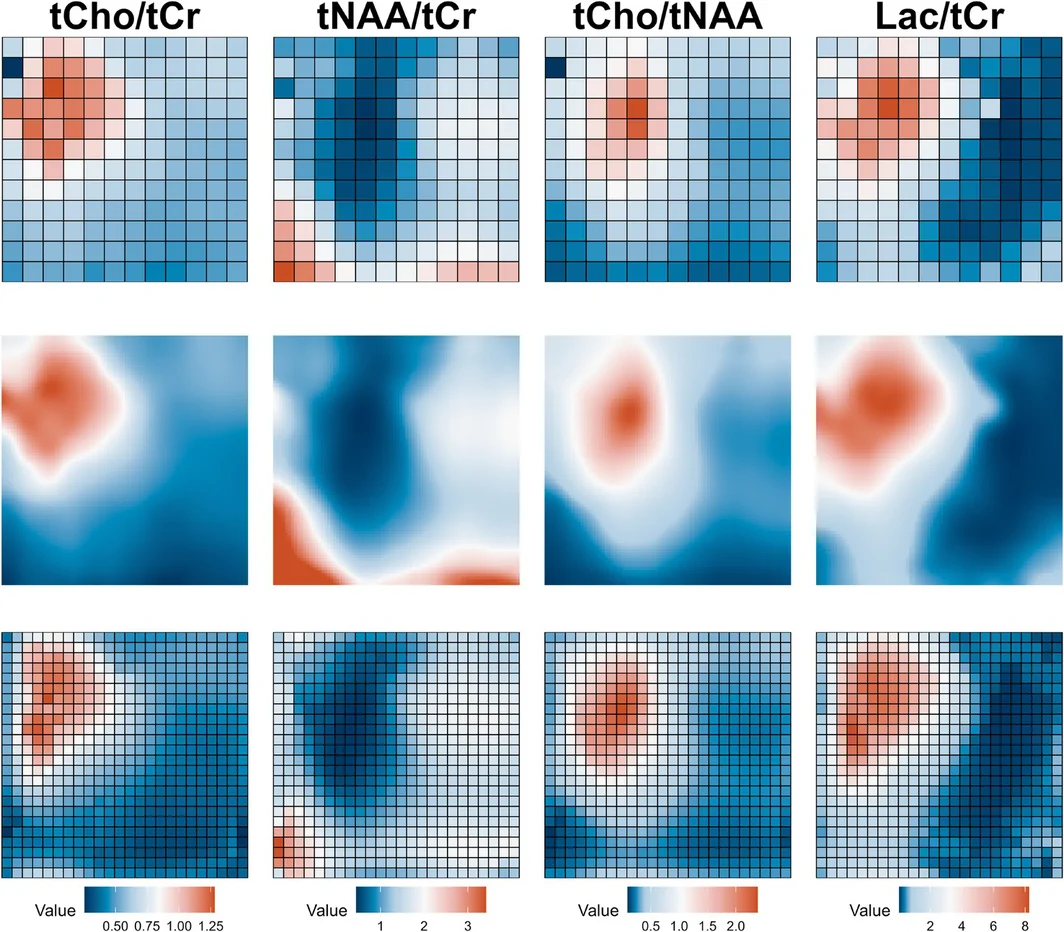
Metabolite ratio grids, corresponding model spatial prediction and metabolite ratio grids obtained after zero‐filling. Colorbar legend is centred (white) on their median value.

Local *R*‐squared maps in Figure 9 indicate that model performance declines at the outer voxels of the grid, particularly in the corners. The number of neighbouring data points around outer voxels is limited. This reduced local sample size can lead to less stable coefficient estimates and increased susceptibility to noise, resulting in the observed decline in goodness‐of‐fit.

**FIGURE 9:**
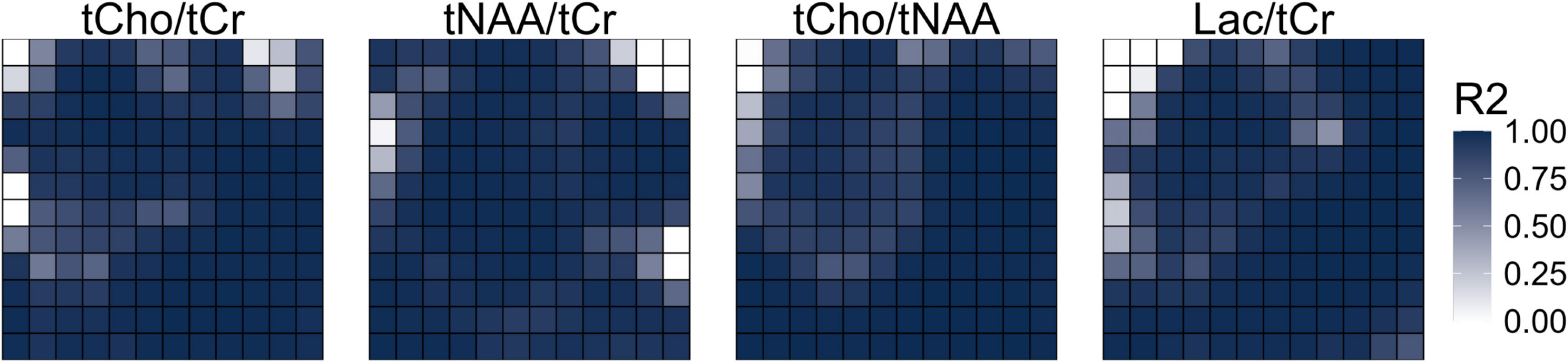
Local *R*‐squared maps for each model using NNGWR.

The spatial distribution of metabolite ratios within brain compartments may also provide new insights. As shown in Figure 10, metabolite distributions within the *Tumour* region are spatially heterogenous. Specifically, in the anterior aspect, we report elevated Lac/tCr ratios and increased tCho relative concentrations with decreased tNAA/tCr ratios, compared with the posterior portion.

**FIGURE 10:**
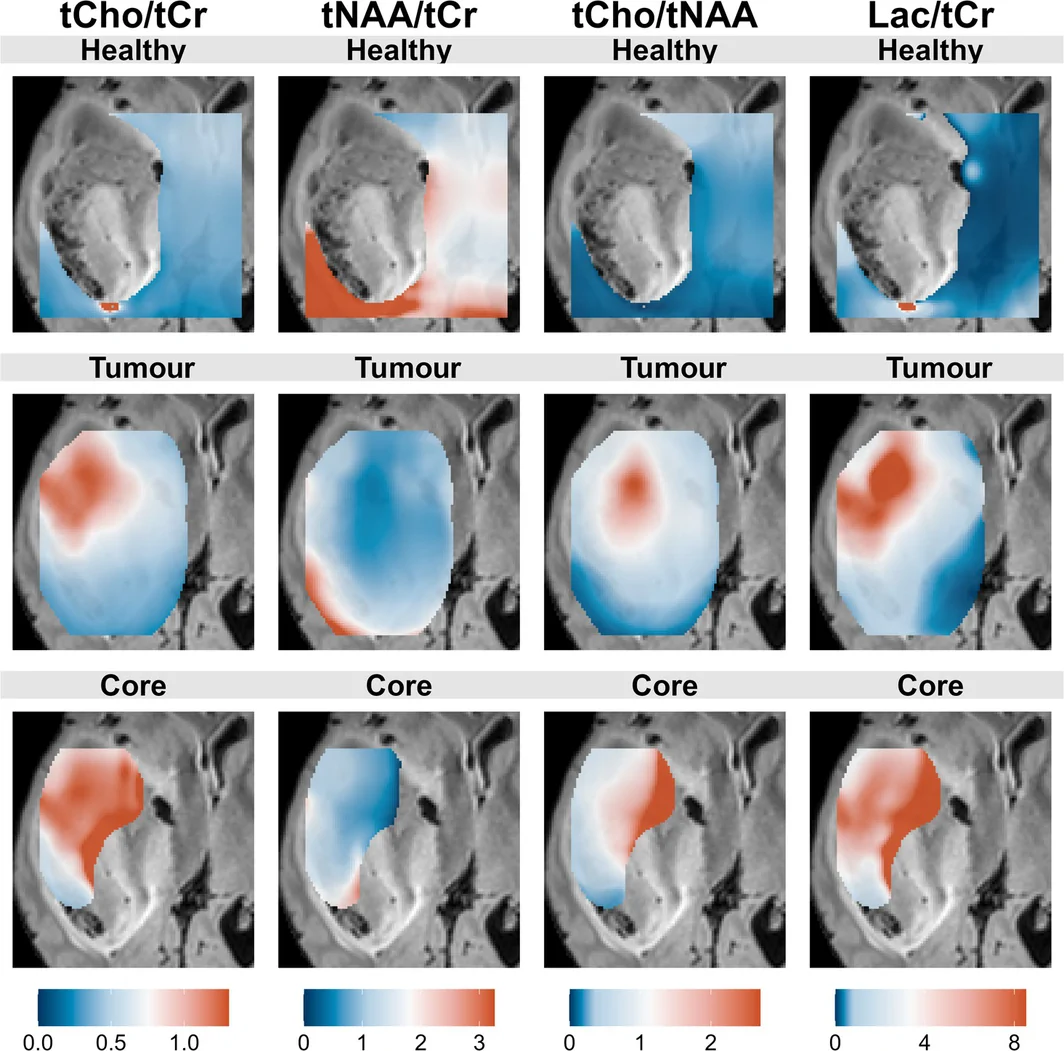
Map of compartment‐specific metabolic ratios estimates. Colorbar legend is centred (white) on metabolite ratios median value. Coefficients at locations where the corresponding tissue rate does not exceed 25% are not displayed.

Separating metabolite ratios by brain compartment enables a comparison of their intrinsic spatial distributions. Notably, Lac/tCr ratios were nearly absent in healthy tissues (0.25±0.49), markedly elevated in tumour regions (2.59±2.99), and highest in core tumour areas (4.18±4.52). tCho/tCr ratio peaked in core tumour (1.10±0.62), with high levels in tumour (0.58±0.34), and were lowest in healthy regions (0.36±0.2). Similarly, tCho/tNAA ratios were highest in core tumour (0.75±0.77), elevated in tumour (0.65±0.58) and remained low in healthy tissues (0.18±0.19). In contrast, tNAA/tCr ratios were predominantly observed in healthy regions (2.40±1.02), with significantly reduced levels in tumour (1.25±0.58) and core tumour (1.52±1.54). Wilcoxon comparison tests show that the median metabolite ratios differ significantly across all brain regions (adjusted *p* < 0.001), except for the tNAA/tCr ratio within the tumour, where no significant difference is observed between the Core and non‐Core regions (Figure 11). To prevent an artificial inflation of degrees of freedom, we restricted our comparisons to estimates localised at voxel centroids.

**FIGURE 11:**
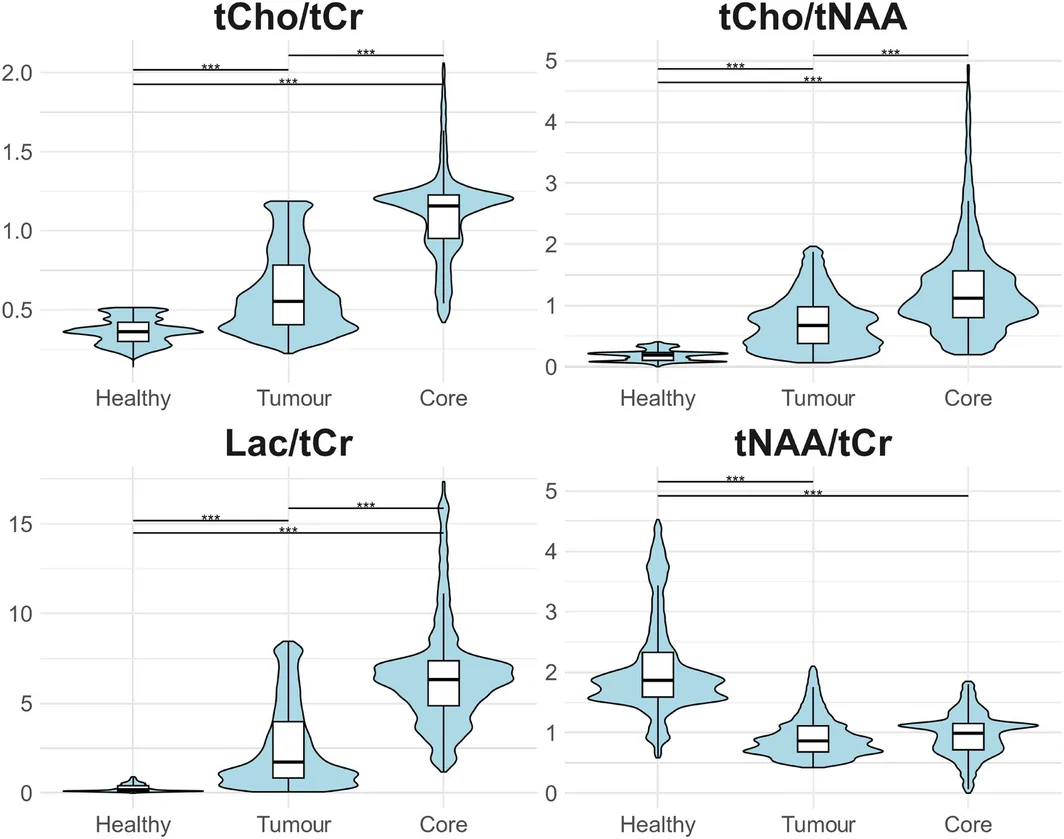
Violin plot of metabolic ratios estimates across brain tissue type with Wilcoxon's test of comparison. All significant *p*‐values are under 0.001 and coefficients for which its corresponding tissue rate does not exceed 25% are removed.

For metabolite ratios, the estimated *R*‐squared obtained are 0.93 for tCho/tNAA ratio, 0.97 for tNAA/tCr ratio, 0.95 for tCho/tCr ratio and 0.94 for Lac/tCr ratio. For metabolites alone, the estimated *R*‐squared are 0.93 for tCho, 0.95 for tNAA, 0.95 for tCr and 0.94 for Lac.

## 5 Discussion

We aimed at improving MRSI spatial information by proposing a statistical framework that interpolates MRSI data across the VOI and separates the contributions of multiple brain compartments. Unlike the SLIM method and its variants, which assume metabolic homogeneity within each compartment [4], this model accounts for metabolic variations to provide a more biologically plausible representation.

In MRSI super‐resolution research, direct ground truth is inherently unavailable [4]. Therefore, we evaluated the model's reliability by assessing its average quality‐of‐fit within a cohort of young healthy volunteers. This analysis was completed with a simulation study, in which the model performances behaviour was evaluated under different acquisitions conditions.

The proposed model outperforms zero‐filling and cubic spline interpolations in simulations, particularly in cases with high noise levels and low grid resolution. This demonstrates that the anatomically consistent interpolation provided by the NNGWR model is crucial for recovering information lost in low‐resolution MRSI. It should be noted that zero‐filling is simply designed to resample the data onto a finer grid without introducing new information [4]. In addition, unlike zero‐filling or spline interpolation, our approach separates the reconstructed maps across anatomical compartments, providing structural information that these standard methods do not capture.

The model showed strong predictive performance for major metabolites, with high *R*‐squared values for tCho, tNAA and tCr. Lac and Lac‐based ratios returned much lower *R*‐squared values, whereas higher levels of lactate were found in the glioblastoma clinical case and the model reached a better quality‐of‐fit. Therefore, the lower *R*‐squared values are likely due to the higher quantification uncertainty resulting from the low concentration of lactate in healthy brain tissue.

We introduced a robust reweighting method to account for variable spectral quality, by downweighting progressively the importance of data associated with unreliable spectra. This method replaces conventional hard‐thresholding [10] with a smoother procedure. In the present work we used a function with compact support and thresholds relative to the observed distribution of quality metrics. Therefore, the data reweighting adapts to the applications and the sequence, and highly degraded spectra may be removed. By design, these weights equalise when spectral quality metrics are uniform, and the model reverts to its standard formulation. This method can be integrated into most regression and optimisation models.

The median and maximum percentage of deviation were greatly reduced, demonstrating that the robust reweighting reduces the susceptibility to spectral degradation while mitigating the risk of estimation failure. Moreover, the clinical case illustrates the model's ability to handle poor‐quality spectra by excluding them. In such scenarios, the spatial prediction is informed by surrounding data, allowing the model to perform data imputation. By neutralising local artefacts and downweighting degraded spectra, the model achieves a more reliable interpolation than standard methods, which would otherwise be biased by such spectral degradations.

In the healthy cohort, we observed lower tCho/tCr and tNAA/tCr ratios in GM compared with WM, consistent with previous findings [30, 31]. Furthermore, the increase of tCho ratios and Lac/tCr ratios, alongside a decrease of tNAA/tCr within the glioblastoma relative to healthy tissue, are concordant with the metabolic alterations characteristic of high‐grade gliomas [11]. Moreover, interpolations of Lac/tCr and tCho ratios are heterogeneously distributed within the glioblastoma. These new spatial indicators could be helpful to predict the tumour propagation direction and to map the heterogeneity of tumour aggressiveness. Additionally, the model's interpolation facilitates retrospective voxel alignment with an ROI.

The bandwidth parameter h was optimised for each metabolite ratio, and its distribution significantly differed across them, indicating that the optimal scale of spatial variation is different between metabolites. However, caution is needed in interpretation, as its estimation is purely data‐driven and may reflect not only underlying biological processes but also technical factors influencing the final MRSI data output.

The partitioning of metabolite ratios by brain compartment facilitates a precise comparison of their average distributions. Although previous methodologies were constrained to target regions dominated by a single tissue type [31, 32], our approach separates these contributions, enabling accurate analysis of segmented ROIs, even within physiologically mixed areas.

Recalling that this is a statistical model, it should be understood as a possible representation of reality, providing intrinsic metabolic distributions within biologically justified compartments, rather than a definitive ground truth. Local statistics, such as the local *R*‐squared act as diagnostic indicators that alert the user to potential spectral artefacts or inaccuracies in the anatomical segmentation. Particular caution is advised when interpreting estimates for metabolites with low concentration in regions where the corresponding tissue compartment is underrepresented. Such edge cases may lead to lower numerical stability.

A further limitation concerns the simulation design. Zero‐filling was applied to downsampled, real‐valued concentration maps rather than to complex k‐space or time‐domain data prior to quantification. Consequently, the simulation study doesn't entirely reflect the in vivo MRSI data acquisition workflow, and the reported errors should be viewed as optimistic estimates. However, our model outperforms zero‐filled interpolation across all tested settings, suggesting that its relative advantage is robust even if absolute error levels in vivo may differ.

In contrast to deep learning‐based super‐resolution methods, our model requires only a single MRSI acquisition and anatomical segmentation, without any training phase or large annotated datasets. This makes it more practical for clinical use. Moreover, MRSI super‐resolution studies often rely on limited or artificial training data, which can lead to reduced performance when models are applied to real‐world acquisitions [33].

We focused this work on metabolite ratios as they are the most common quantification method in clinical settings [16]. The current model is not directly applicable to absolute quantification, as this would require accounting for partial volume effects, relaxation time effects [15, 34] and further validation. Finally, although we applied this model to phase‐encoded MRSI, it can be adapted to other localisation techniques, such as 3D echo‐planar spectroscopic imaging [30], as long as an appropriate definition of the SRF is provided.

More appropriate GWR models could also be used to address specific study objectives or clinical applications. For instance, mixed GWR could be used to enforce metabolic homogeneity in certain compartments [35], whereas multiscale GWR would allow for greater spatial heterogeneity in targeted regions. Additionally, brain tissue anisotropy could be accounted for by using anisotropic kernels in the distance‐weighting matrix (9).

Given that this model is applicable regardless of magnetic field strength, incorporating CSDE could enable a more precise characterisation of MRS data. This would improve performance particularly at high magnetic fields, where CSDE are more pronounced.

## 6 Conclusions

We introduced a novel statistical framework based on NNGWR that interpolates the metabolite ratios and estimate compartment‐specific contributions in the presence of partial volume effects. This model allows each compartment to exhibit spatial heterogeneity, leading to a more physiologically realistic representation of brain metabolism. Real‐world MRSI data is managed through a robust reweighting scheme that mitigates the influence of spatially varying spectral quality and improves model stability.

When applied to a glioblastoma clinical case, the framework successfully separated metabolite ratios across healthy, tumour and necrotic tissues. It enhanced MRSI data interpretability by providing multiple local and global indicators, such as *R*‐squared maps which offer transparency by identifying regions of lower fit quality. Given this open framework and adaptability, this model is potentially applicable in both clinical and research settings.

## Author Contributions

**Clément Giraud:** conceptualisation, data curation, formal analysis, software, validation, methodology, investigation, visualisation, writing – original draft, writing – review and editing. **Arnaud Poinas:** conceptualisation, formal analysis, investigation, validation, supervision, writing – review and editing. **Carole Guillevin:** conceptualisation, data curation, investigation, writing – review and editing. **Rémy Guillevin:** supervision, resources, project administration, writing – review and editing.

## Funding

The authors have nothing to report.

## Conflicts of Interest

The authors declare no conflicts of interest.

## Supporting Information

Additional supporting information may be found in the online version of the article at the publisher's website.

## Figures and Tables

**FIGURE C1:**
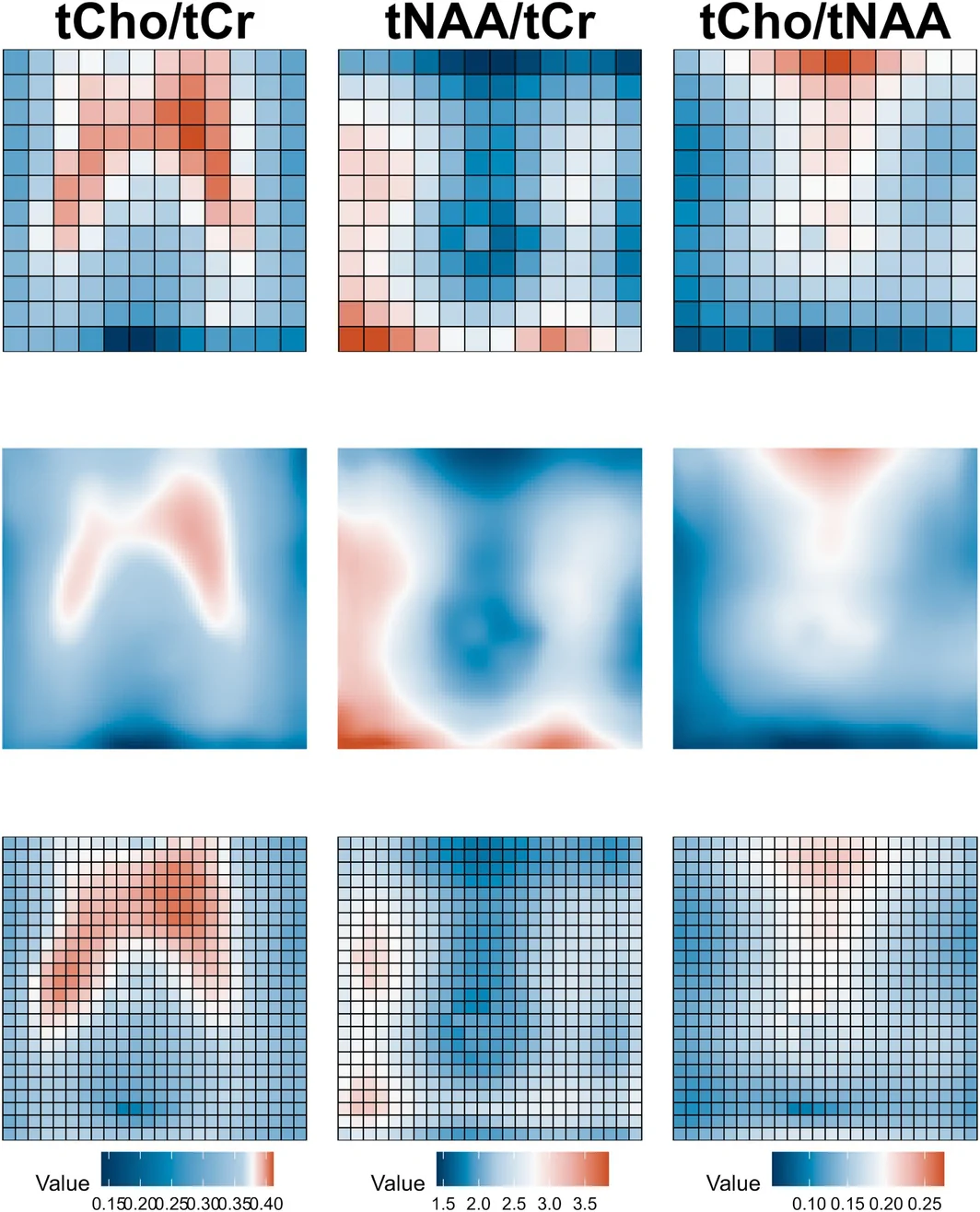
Healthy case: metabolite ratio grids, corresponding model spatial prediction and metabolite ratio grids obtained after zero‐filling. Colorbar legend is centred (white) on their median value.

**FIGURE C2:**
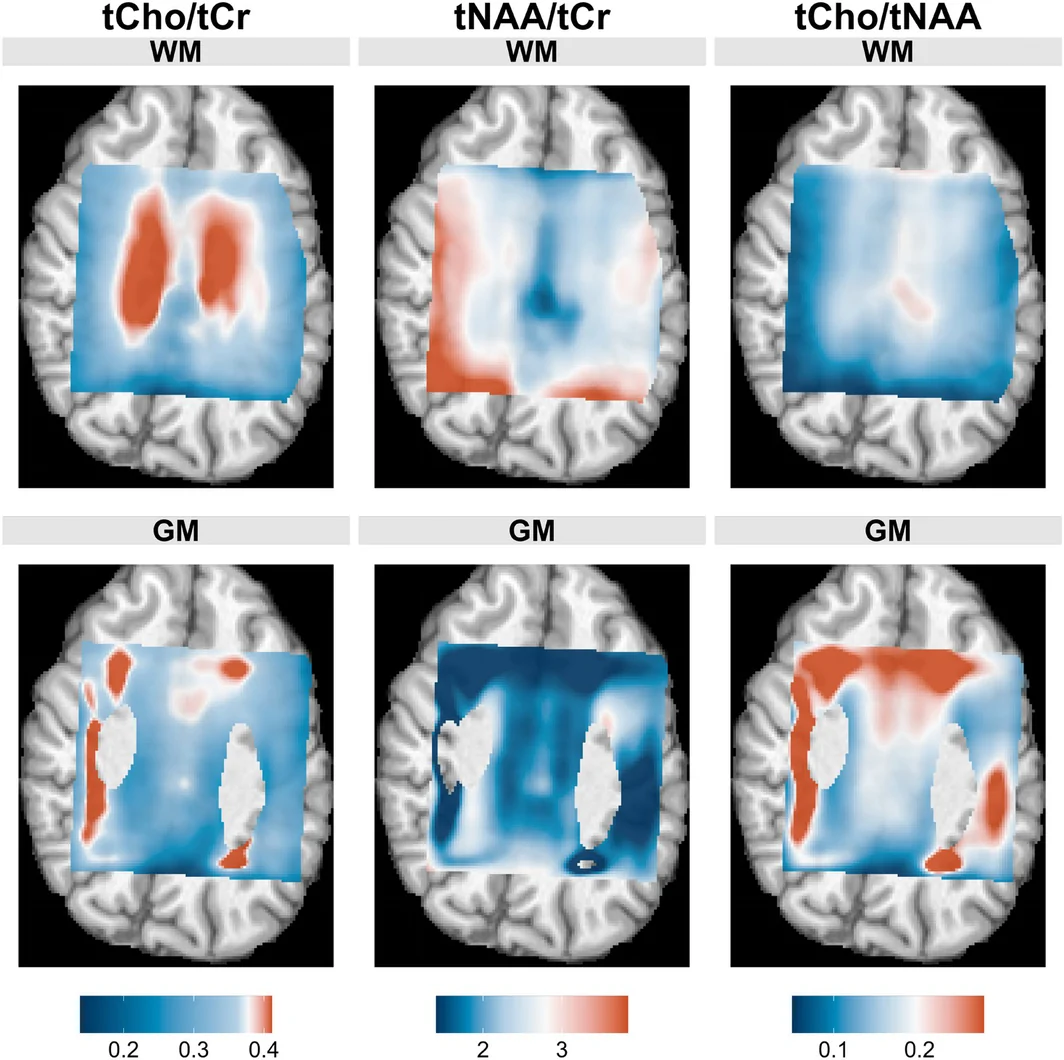
Healthy case: map of metabolic ratios estimates. Colorbar legend is centred (white) on metabolite ratios median value. Coefficients at locations where the corresponding tissue rate does not exceed 25% are not displayed.

**FIGURE C3:**
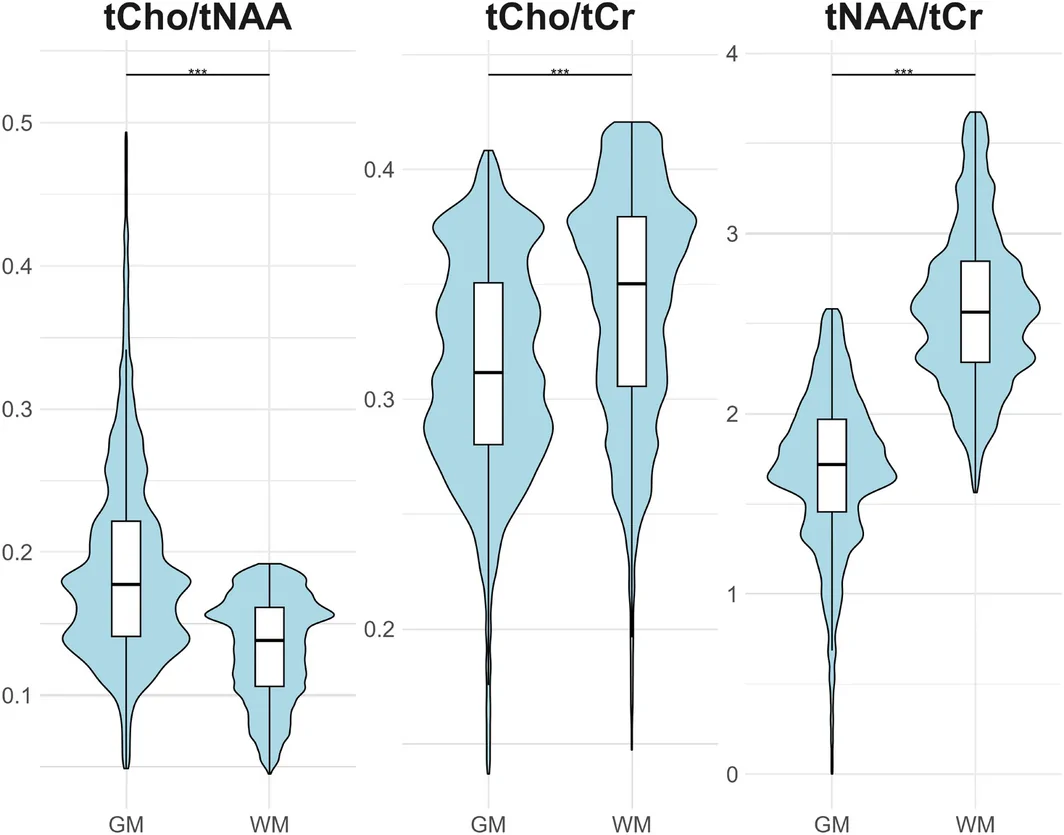
Healthy case: violin plot of metabolic ratios estimates across brain GM and WM with Wilcoxon's test of comparison. Coefficients for which its corresponding tissue rate does not exceed 25% are removed.

## Data Availability

Research data are not shared.

## Appendix A Approximation of Convolution Ratios

### A.1

Proposition 1 Let Ω be a bounded set of ${\mathbb{R}}^{d}$ for some d≥1,h an integrable function on Ω, and f,g be two functions with support on Ω that are also twice continuously differentiable on Ω. Then, there exists a constant C>0 such that for all x∈Ω,

(A1)
$$\begin{array}{cc} & \left|\frac{f*h(x)}{g*h(x)}-\frac{\frac{f}{g}*h(x)}{1_{\Omega }*h(x)}\right|\\ & \leq \frac{1}{g*h(x)1_{\Omega }*h(x)}\left(\left\|\left(\nabla \frac{f}{g}\right)(x)\right\|\|\nabla g(x)\|\|c(x){\|}^{2}+CV(x)\right), \end{array}$$

where $1_{\Omega }$ is the indicator function over Ω,

$$c(x)=\int_{\;\Omega }h(x-y)(x-y)dy$$

and

$$V(x)=\int_{\;\Omega }h(x-y)\|x-y{\|}^{2}dy.$$

Identity (A1) shows that the approximation of the convolution ratio $\frac{f*h}{g*h}$ by $\frac{\frac{f}{g}*h}{1_{\Omega }*h}$ at location x is most precise when the following conditions are met:

- $\frac{f}{g}$ and g are slowly varying in a neighbourhood of x, yielding small values for $\left\|\left(\nabla \frac{f}{g}\right)(x)\right\|$ and ‖∇g(x)‖.
- g does not have values too close to 0 in a neighbourhood of x, yielding large values for g∗h(x).
- x is far from the boundary of Ω and h is close to a Dirac function, yielding small values for V(x) and ‖c(x)‖ and large values for $1_{\Omega }*h(x)$.

Let x,y∈Ω. Using a first order Taylor approximation on f,g and $\frac{f}{g}$ we get that,

(A2)
$$\left\{\begin{array}{l} f(y)=f(x)+\langle y-x,\nabla f(x)\rangle +\|y-x{\|}^{2}O(1)\\ g(y)=g(x)+\langle y-x,\nabla g(x)\rangle +\|y-x{\|}^{2}O(1) \end{array}\right.$$

and

(A3)
$$\frac{f(y)}{g(y)}=\frac{f(x)}{g(x)}+\frac{1}{g(x)}\langle y-x,\nabla f(x)\rangle -\frac{f(x)}{g{\left(x\right)}^{2}}\langle y-x,\nabla g(x)\rangle +\|y-x{\|}^{2}O(1),$$

where O(1) denotes a bounded function on Ω. Using (A2), g∗h(x) can be expanded into

(A4)
$$\begin{array}{ll} & \int_{\;\Omega }\left(g(x)+\langle y-x,\nabla g(x)\rangle +\|y-x{\|}^{2}O(1)\right)h(x-y)dy\;\\ & \;=g(x)1_{\Omega }*h(x)-\langle c(x),\nabla g(x)\rangle +V(x)O(1),\; \end{array}$$

with a similar expression for f∗h(x). Using (A3) and applying the same reasoning leads to

(A5)
$$\begin{array}{cc} \frac{f}{g}*h(x) & =\frac{f(x)}{g(x)}1_{\Omega }*h(x)\\ & -\frac{1}{g(x)}\langle c(x),\nabla f(x)\rangle +\frac{f(x)}{g{\left(x\right)}^{2}}\langle c(x),\nabla g(x)\rangle +V(x)O(1). \end{array}$$

Finally, combining (A4) and (A5) we get, after some computations,

$$\begin{array}{l} \left(\frac{f}{g}*h(x)\right)\left(g*h(x)\right)-f*h(x)1_{\Omega }*h(x)\;\\ =\frac{1}{g(x)}\langle c(x),\nabla f(x)\rangle \langle c(x),\nabla g(x)\rangle -\frac{f(x)}{g{\left(x\right)}^{2}}{\left\langle c(x),\nabla g(x)\right\rangle }^{2}+V(x)O(1)\;\\ =\left\langle c(x),\left(\nabla \frac{f}{g}\right)(x)\right\rangle \langle c(x),\nabla g(x)\rangle +V(x)O(1).\; \end{array}$$

Applying the Cauchy–Schwarz inequality to $\left\langle c(x),\left(\nabla \frac{f}{g}\right)(x)\right\rangle$ and ⟨c(x),∇g(x)⟩ results in the desired bound (A1).

## Appendix B MRS Protocol Details

### B.1

**TABLE B1:** MRS protocol details.

Hardware	
a. Field strength	3T
b. Manufacturer	Siemens
c. Model (software version if available)	Skyra
d. RF coils: nuclei (transmit/receive), number of channels, type, body part	64 channel ^1^H head coil
Acquisition	
Item	Details
a. Pulse sequence	2D CSI SLASER
b. VOI locations	The VOI for the healthy cohort were localised at the level of the basal ganglia and semioval centre (two sequences).
c. Nominal VOI and voxel size	VOI:82.5×82.5×20 mm^3^, voxel: 6.875×6.875×20 mm^3^
d. Repetition time (TR), echo time (TE)	TR 1700 ms, TE 135 ms
e. Total number of excitations or acquisitions per spectrum	One acquisition
f. Additional sequence parameters	Spectral width: 1200 Hz, number of spectral points: 1024, FOV: 220×220×20 mm^3^, matrix size: 32×32, phase‐encoding matrix size: 12×12, no acceleration factor, zero‐filling factor: 2, sampling method: rectilinear, k‐space filtering: Hamming
g. Water suppression method	CHESS, water suppression pulses 50 Hz
h. Shimming method, reference peak and thresholds for acceptance	Automated B0 field mapping
i. Triggering or motion correction method	Motion artefacts were investigated, and none were found.
Data analysis methods and outputs	
a. Analysis software	LCModel
b. Processing steps deviating from quoted reference or product	No deviations took place in the pre‐processing steps from Provencher (2021).
c. Output measure	Ratios to total creatine (creatine + phosphocreatine) and total choline (phosphocholine + glycerophosphocholine) over total NAA (*N*‐acetylaspartate + *N*‐acetylaspartylglutamate)
d. Quantification references and assumptions	Basis set included spectra of alanine, aspartate, creatine, phosphocreatine, GABA, glucose, glutamine, glutathione, myo‐inositol, lactate, tNAA, tCho, phosphoethanolamine, scyllo‐inositol and taurine
Data quality	
a. Reported variables	Metabolite ratios, SNR and FWHM reported by LCModel.
b. Data exclusion criteria	Every spectrum is included in the proposed model. A reweighted strategy is applied prior to spectral quality.
c. Quality measures of postprocessing model fitting	See above.
d. Sample spectrum	Figure 6

## Appendix C Application of the Model to a Healthy Case

### C.1

To illustrate the model's application in the healthy cohort, we present the results obtained for a selected subject (Figure C1). We modelled the tNAA/tCr, tCho/tCr and tCho/tNAA ratios. The estimated *R*‐squared values for this case were 0.91 for the tNAA/tCr ratio, 0.84 for the tCho/tCr ratio and 0.89 for the tCho/tNAA ratio.

The estimated metabolite ratio maps are shown in Figure C2, and the metabolic distribution across brain compartments is displayed in Figure C3.
